# An in-depth evaluation of federated learning on biomedical natural language processing for information extraction

**DOI:** 10.1038/s41746-024-01126-4

**Published:** 2024-05-15

**Authors:** Le Peng, Gaoxiang Luo, Sicheng Zhou, Jiandong Chen, Ziyue Xu, Ju Sun, Rui Zhang

**Affiliations:** 1 Department of Computer Science and Engineering, University of Minnesota, Minneapolis, MN USA; 2 Department of Computer and Information Science, University of Pennsylvania, Philadelphia, PA USA; 3 Institute for Health Informatics, University of Minnesota, Minneapolis, MN USA; 4 Nvidia Corporation, Santa Clara, CA USA; 5 Division of Computational Health Sciences, Department of Surgery, University of Minnesota, Minneapolis, MN USA

**Keywords:** Machine learning, Data mining

## Abstract

Language models (LMs) such as BERT and GPT have revolutionized natural language processing (NLP). However, the medical field faces challenges in training LMs due to limited data access and privacy constraints imposed by regulations like the Health Insurance Portability and Accountability Act (HIPPA) and the General Data Protection Regulation (GDPR). Federated learning (FL) offers a decentralized solution that enables collaborative learning while ensuring data privacy. In this study, we evaluated FL on 2 biomedical NLP tasks encompassing 8 corpora using 6 LMs. Our results show that: (1) FL models consistently outperformed models trained on individual clients’ data and sometimes performed comparably with models trained with polled data; (2) with the fixed number of total data, FL models training with more clients produced inferior performance but pre-trained transformer-based models exhibited great resilience. (3) FL models significantly outperformed pre-trained LLMs with few-shot prompting.

## Introduction

The recent advances in deep learning have sparked the widespread adoption of language models (LMs), including prominent examples of BERT^1^ and GPT^2^, in the field of natural language processing (NLP). These LMs are trained on massive amounts of public text data, comprising billions of words, and have emerged as the dominant technology for various linguistic tasks, including text classification^3,4^, text generation^5,6^, information extraction^7–9^, and question answering^10,11^. The success of LMs can be largely attributed to their ability to leverage large volumes of training data. However, in privacy-sensitive domains like medicine, data are often naturally distributed, making it difficult to construct large corpora to train LMs. To tackle the challenge, the most common approach thus far has been to fine-tune pre-trained LMs for downstream tasks using limited annotated data^12,13^. Nevertheless, pre-trained LMs are typically trained on text data collected from the general domain, which exhibits divergent patterns from that in the biomedical domain, resulting in a phenomenon known as domain shift. Compared to general text, biomedical texts can be highly specialized, containing domain-specific terminologies and abbreviations^14^. For example, medical records and drug descriptions often include specific terms that may not be present in general language corpora, and the terms often vary among different clinical institutes. Also, biomedical data lacks uniformity and standardization across sources, making it challenging to develop NLP models that can effectively handle different formats and structures. Electronic Health Records (EHRs) from different healthcare institutions, for instance, can have varying templates and coding systems^15^. So, direct transfer learning from LMs pre-trained on the general domain usually suffers a drop in performance and generalizability when applied to the medical domain as is also demonstrated in the literature^16^. Therefore, developing LMs that are specifically designed for the medical domain, using large volumes of domain-specific training data, is essential. Another vein of research explores pre-training the LM on biomedical data, e.g., BlueBERT^12^ and PubMedBERT^17^. These LMs were either pre-trained on mixed-domain data (first pre-train on the general text and then keep pre-train on biomedical text) or directly pre-trained on domain-specific public medical datasets, e.g., PubMed literature and the Medical Information Mart for Intensive Care (MIMIC III)^18^ and have shown improved performances compared to classical methods such as conditional random field (CRF)^19^ and recurrent neural network (RNN) (e.g., long-short-term memory (LSTM)^20^) in many biomedical text mining tasks^8,9,12,16,21^. Nonetheless, it is important to highlight that the efficacy of these pre-trained medical LMs heavily relies on the availability of large volumes of task-relevant public data, which may not always be readily accessible.

All these mentioned above represent the classical *centralized learning* regime, which involves aggregating data from distributed data sites and training a model in a single environment. However, this approach poses significant challenges in medicine, where data privacy is crucial and data access is restricted due to regulatory concerns. Thus, in practice, people can only perform training with local datasets—*single-client training*. The drawback comes when the local dataset is small and often gives poor performance when evaluating an external dataset—poor generalization. To take advantage of the massively distributed data as well as improve the model generalizability, federated learning (FL) was initialized in 2016^22^ as a novel learning scheme to empower training with a decentralized environment and achieve many successes in critical domains with data privacy restriction^23–25^. In an FL training loop, clients jointly train a shared global model by sharing the model weights or gradients while keeping their data stored locally. By bringing the model to the data, FL strictly ensures data privacy while achieving competitive levels of performance compared to a model trained with pooled data. While there is a rise of research showing great promise of applying FL in general NLP^26,27^, applications of FL in biomedical NLP are still under-explored. Existing works in FL on biomedical NLP are either focused on optimizing one task^28,29^ or trying to improve communication efficiency^28^. The current literature lacks a comprehensive comparison of FL on varied biomedical NLP tasks with real-world perturbations. To close this gap, we conducted an in-depth study of two representative NLP tasks, i.e., named entity recognition (NER) and relation extraction (RE), to evaluate the feasibility of adopting FL (e.g., FedAvg^30^ and FedProx^31^) with LMs (e.g., Transformer-based models) in biomedical NLP. Our study aims to provide an in-depth investigation of FL in biomedical NLP by studying several FL variants on multiple practical learning scenarios, including varied federation scales, different model architectures, data heterogeneities, and comparison with large language models (LLMs) on multiple benchmark datasets. Our major findings include:

1. When data were independent and identically distributed (IID), models trained using FL, especially pre-trained BERT-based models, performed comparable to centralized learning, a significant boost to single-client learning. Even when data were non-IID distributed, the gap can be filled by using alternative FL algorithms.
2. Larger models exhibited better resistance to the changes in FL scales. With a fixed number of data, the performance of FL models overall degraded as the clients’ size increased. However, the deterioration diminished when combined with larger pre-trained models such as BERT-based models and GPT-2.
3. FL significantly outperformed pre-trained LLMs, e.g., GPT-4, PaLM 2, and Gemini Pro, with few-shot prompting.

## Results

In this section, we present our main results of analysis on FL with a focus on several practical facets, including (1) learning tasks, (2) scalability, (3) data distribution, (4) model architectures and sizes, and (5) comparative assessments with LLMs.

### FedAvg, single-client, and centralized learning for NER and RE tasks

Table 1 offers a summary of the performance evaluations for FedAvg, single-client learning, and centralized learning on five NER datasets, while Table 2 presents the results on three RE datasets. Our results on both tasks consistently demonstrate that FedAvg outperformed single-client learning. Notably, in cases involving large data volumes, such as BC4CHEMD and 2018 n2c2, FedAvg managed to attain performance levels on par with centralized learning, especially when combined with BERT-based pre-trained models.

**Table 1:** Comparison of FedAvg with centralized learning and single-client learning on 5 NER tasks measured by F1-score with lenient (upper) and strict (lower, inside parenthesis) matching scheme

Model	Method	2018 n2c2	BC2GM	BC4CHEMD	JNLPBA	NCBI-disease
BERT	Centralized	0.879 ± 0.002 (0.822 ± 0.001)	0.972 ± 0.001 (0.928 ± 0.001)	0.981 ± 0.001 (0.968 ± 0.001)	0.969 ± 0.001 (0.939 ± 0.002)	0.989 ± 0.001 (0.973 ± 0.000)
	Single (avg)	0.828 ± 0.003 (0.761 ± 0.007)	0.888 ± 0.003 (0.759 ± 0.003)	0.921 ± 0.002 (0.881 ± 0.002)	0.905 ± 0.002 (0.815 ± 0.003)	0.917 ± 0.004 (0.847 ± 0.008)
	FedAvg	**0.877** ± **0.002** (0.817 ± 0.002)	0.959 ± 0.001 (0.897 ± 0.000)	0.973 ± 0.000 (0.954 ± 0.001)	0.949 ± 0.001 (0.896 ± 0.001)	0.976 ± 0.001 (0.949 ± 0.001)
BlueBERT	Centralized	0.879 ± 0.005 (0.820 ± 0.007)	0.975 ± 0.000 (0.932 ± 0.002)	0.965 ± 0.004 (0.944 ± 0.007)	0.969 ± 0.001 (0.940 ± 0.003)	0.987 ± 0.008 (0.968 ± 0.009)
	Single (avg)	0.823 ± 0.043 (0.753 ± 0.047)	0.906 ± 0.003 (0.778 ± 0.004)	0.927 ± 0.003 (0.890 ± 0.004)	0.908 ± 0.002 (0.818 ± 0.003)	0.925 ± 0.004 (0.855 ± 0.009)
	FedAvg	**0.876** ± **0.002 (0.817** ± **0.000)**	0.966 ± 0.001 (0.919 ± 0.002)	**0.977** ± **0.000 (0.959** ± **0.000)**	0.963 ± 0.001 (0.923 ± 0.001)	**0.984** ± **0.002 (0.963** ± **0.000)**
BiLSTM-CRF	Centralized	0.834 ± 0.002 (0.783 ± 0.002)	0.924 ± 0.001 (0.866 ± 0.001)	0.958 ± 0.001 (0.934 ± 0.001)	0.961 ± 0.000 (0.924 ± 0.001)	0.971 ± 0.002 (0.944 ± 0.004)
	Single (avg)	0.729 ± 0.004 (0.663 ± 0.004)	0.622 ± 0.006 (0.415 ± 0.006)	0.766 ± 0.002 (0.673 ± 0.003)	0.822 ± 0.002 (0.670 ± 0.005)	0.733 ± 0.010 (0.589 ± 0.012)
	FedAvg	0.782 ± 0.002 (0.734 ± 0.003)	0.793 ± 0.005 (0.645 ± 0.013)	0.920 ± 0.002 (0.882 ± 0.002)	0.902 ± 0.001 (0.810 ± 0.004)	0.865 ± 0.020 (0.767 ± 0.035)
BioBERT	Centralized	0.884 ± 0.002 (0.823 ± 0.002)	0.980 ± 0.000 (0.937 ± 0.003)	0.983 ± 0.001 (0.972 ± 0.001)	0.971 ± 0.000 (0.943 ± 0.001)	0.993 ± 0.001 (0.975 ± 0.001)
	Single (avg)	0.845 ± 0.003 (0.780 ± 0.007)	0.931 ± 0.002 (0.808 ± 0.002)	0.944 ± 0.002 (0.912 ± 0.002)	0.915 ± 0.002 (0.829 ± 0.003)	0.936 ± 0.004 (0.869 ± 0.006)
	FedAvg	0.879 ± 0.002 **(0.818** ± **0.003)**	0.974 ± 0.001 (0.922 ± 0.000)	0.978 ± 0.000 (0.963 ± 0.001)	0.957 ± 0.001 (0.910 ± 0.002)	0.983 ± 0.002 (0.958 ± 0.001)
Bio_clincialBERT	Centralized	0.885 ± 0.006 (0.827 ± 0.005)	0.974 ± 0.001 (0.933 ± 0.001)	0.980 ± 0.001 (0.967 ± 0.001)	0.969 ± 0.001 (0.941 ± 0.001)	0.993 ± 0.001 (0.975 ± 0.001)
	Single (avg)	0.828 ± 0.003 (0.761 ± 0.007)	0.898 ± 0.003 (0.768 ± 0.004)	0.924 ± 0.001 (0.886 ± 0.002)	0.905 ± 0.002 (0.815 ± 0.003)	0.922 ± 0.005 (0.853 ± 0.008)
	FedAvg	**0.878** ± **0.001** (0.815 ± 0.001)	0.960 ± 0.002 (0.901 ± 0.001)	0.971 ± 0.001 (0.953 ± 0.001)	0.951 ± 0.000 (0.901 ± 0.001)	0.982 ± 0.003 (0.958 ± 0.004)
GPT-2	Centralized	0.801 ± 0.001 (0.745 ± 0.001)	0.891 ± 0.001 (0.836 ± 0.001)	0.879 ± 0.002 (0.857 ± 0.002)	0.925 ± 0.001 (0.881 ± 0.001)	0.928 ± 0.002 (0.904 ± 0.002)
	Single (avg)	0.741 ± 0.005 (0.681 ± 0.005)	0.714 ± 0.005 (0.554 ± 0.005)	0.747 ± 0.003 (0.687 ± 0.004)	0.798 ± 0.004 (0.672 ± 0.005)	0.767 ± 0.004 (0.690 ± 0.010)
	FedAvg	**0.798** ± **0.003 (0.746** ± **0.001)**	0.796 ± 0.001 (0.674 ± 0.006)	0.825 ± 0.000 (0.794 ± 0.000)	0.844 ± 0.001 (0.748 ± 0.001)	0.852 ± 0.003 (0.809 ± 0.002)

For datasets involving multiple entities, we report the macro average score. The reported values represent the mean and standard deviation over three repeated experiments^a^.

^a^FedAvg that matched (with overlapped intervals) or surpassed the centralized learning of the same model are bolded and the highest scores for each corpus are underlined.

**Table 2:** Comparison of FedAvg with centralized learning and single-client learning on RE task measure by macro F1-score

Model	Method	2018 n2c2	EUADR	GAD
BERT	Centralized	0.947 ± 0.001	0.750 ± 0.040	0.738 ± 0.028
	Single (avg)	0.892 ± 0.007	0.522 ± 0.111	0.642 ± 0.017
	FedAvg	**0.946** ± **0.002**	0.527 ± 0.008	**0.703** ± **0.021**
BlueBERT	Centralized	0.950 ± 0.002	0.582 ± 0.109	0.755 ± 0.007
	Single (avg)	0.898 ± 0.020	0.452 ± 0.039	0.616 ± 0.030
	FedAvg	**0.950** ± **0.002**	**0.548** ± **0.073**	0.714 ± 0.018
BioBERT	Centralized	0.942 ± 0.002	0.737 ± 0.049	0.783 ± 0.007
	Single (avg)	0.901 ± 0.006	0.525 ± 0.094	0.684 ± 0.015
	FedAvg	**0.942** ± **0.002**	**0.718** ± **0.037**	0.750 ± 0.008
Bio_ClincialBERT	Centralized	0.950 ± 0.001	0.741 ± 0.067	0.743 ± 0.014
	Single (avg)	0.904 ± 0.006	0.514 ± 0.101	0.623 ± 0.018
	FedAvg	0.946 ± 0.003	0.578 ± 0.057	0.695 ± 0.009
GPT-2	Centralized	0.951 ± 0.004	0.684 ± 0.097	0.709 ± 0.004
	Single (avg)	0.899 ± 0.009	0.468 ± 0.105	0.630 ± 0.017
	FedAvg	**0.946** ± **0.003**	**0.547** ± **0.086**	**0.721** ± **0.009**

The reported values represent the mean and standard deviation over three repeated experiments^a^.

^a^FedAvg that matched (with overlapped intervals) or surpassed the centralized learning of the same model are bolded and the highest scores for each corpus are underlined.

### Influence of FL scale on the performance of LMs

**Fig. 1:**
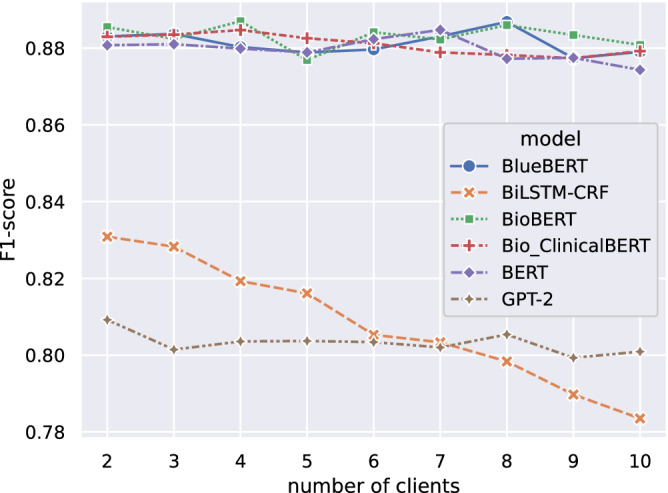
Performance of FL models with varying numbers of clients. We tested models on 2018 n2c2 (NER) and evaluated them using the F1 score with lenient matching scheme.

In clinical applications, there are two distinct learning paradigms. The first involves small-scale client cohorts, each equipped with substantial data resources, often seen in collaborations within hospital networks. In contrast, the second encompasses widely distributed clients, characterized by more limited data holders, often associated with collaborations within clinical facilities or on mobile platforms. We investigated the performance of FL on the two learning paradigms by varying client group sizes while maintaining a fixed total training data volume. The results are summarized in Fig. 1, revealing a consistent trend: notably, larger models, such as those backed by BERT and GPT-2 architectures, exhibited great resilience to fluctuations in federation scales. In contrast, the lightweight model, as of BiLSMT-CRF, was susceptible to alterations of scale, resulting in a rapid deterioration in performance as the number of participating clients increased.

### Comparison of FedAvg and FedProx with data heterogeneity

Biomedical texts often exhibit high specialization due to distinct protocols employed by different hospitals when generating medical records, resulting in great variations—sublanguage differences. Therefore, FL practitioners should account for such data heterogeneity when implementing FL in healthcare systems. We simulated a real non-IID scenario by emulating BC2GM and JNLPBA as two clients and jointly performing FL. We considered two FL algorithms including FedAvg and FedProx; both are widely deployed in practice. For comparison, we also studied a simulated IID setting using the 2018 n2c2 dataset by random splitting. Detailed analysis of the non-IID/IID distribution can be found in Supplementary Fig. 1 and Supplementary Table 3. As shown in Table 3, we observed that the performance of FedProx was sensitive to the choice of the hyper-parameter *μ*. Notably, a smaller *μ* consistently resulted in improved performance. When *μ* was carefully selected, FedProx outperformed FedAvg when the data were non-IID distributed (lenient F1 score of 0.994 vs. 0.934 and strict F1 score of 0.901 vs. 0.884). However, the difference between the two algorithms was mostly indistinguishable when the data were IID distributed (lenient F1 score of 0.880 vs. 0.879 and strict F1 score of 0.820 vs. 0.818).

**Table 3:** Comparison of FedAvg with centralized learning and single-client learning using BioBERT

Method	*µ*	IID (2018 n2c2)		non-IID (BC2GM & JNLPBAS)	
		lenient	strict	lenient	strict
Centralized	–	0.884 ± 0.002	0.823 ± 0.002	0.964 ± 0.001	0.929 ± 0.000
FedAvg	–	0.879 ± 0.002	0.818 ± 0.003	0.934 ± 0.003	0.884 ± 0.003
FedProx	1	0.855 ± 0.003	0.790 ± 0.005	0.880 ± 0.001	0.772 ± 0.002
	0.5	0.868 ± 0.001	0.809 ± 0.002	0.881 ± 0.002	0.777 ± 0.001
	0.1	0.872 ± 0.003	0.814 ± 0.004	0.897 ± 0.002	0.817 ± 0.002
	0.01	0.878 ± 0.003	0.819 ± 0.002	0.933 ± 0.002	0.884 ± 0.003
	0.001	**0.880** ± **0.002**	**0.820** ± **0.001**	0.944 ± 0.002	0.901 ± 0.002

We select the value of *μ* (a hyper-parameter in FedProx) as suggested by the FedProx paper. The reported values represent the mean and standard deviation over three repeated experiments^a^.

^a^FedAvg that matched (with overlapped intervals) or surpassed the centralized learning of the same model are bolded and the highest scores for each corpus are underlined.

### Impact of the LM size on the performance of different training schemes

**Fig. 2:**
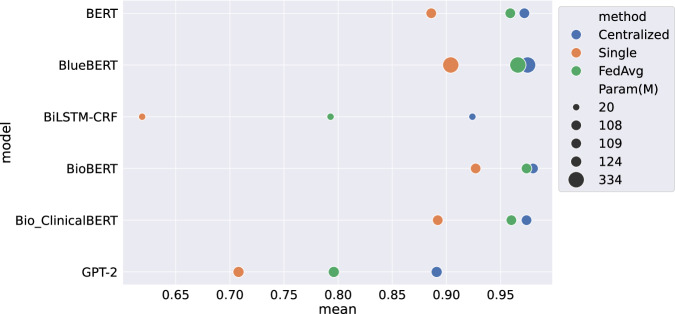
Comparison of model performance with different sizes, measured by the number of trainable parameters on the BC2GM dataset. The size of the circle tells the number of model parameters, while the color indicates different learning methods. The *x*-axis represents the mean test F1-score with the lenient match (results are adapted from Table 1).

We investigated the impact of model size on the performance of FL. We compared 6 models with varying sizes, with the smallest one comprising 20 M parameters and the largest one comprising 334 M parameters. We picked the BC2GM dataset for illustration and anticipated similar trends would hold for other datasets as well. As shown in Fig. 2, in most cases, larger models (represented by large circles) overall exhibited better test performance than their smaller counterparts. For example, BlueBERT demonstrated uniform enhancements in performance compared to BiLSTM-CRF and GPT2. Among all the models, BioBERT emerged as the top performer, whereas GPT-2 gave the worst performance.

### Comparison between FL and LLM

**Fig. 3:**
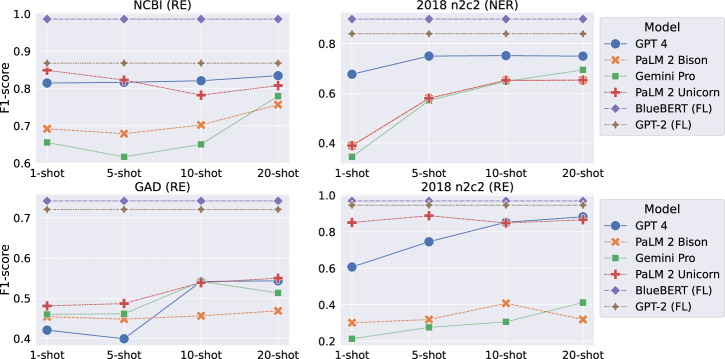
Comparison of LLMs using few-shot prompting and small LMs (BlueBERT and GPT-2) trained with FL on NER (upper) and RE (lower) tasks evaluated based on the F1-score (lenient matching for NER tasks). A complete evaluation, including the strict matching and running time analysis, can be found in Supplementary Table 1 and Supplementary Table 2.

In light of the well-demonstrated performance of LLMs on various linguistic tasks, we explored the performance gap of LLMs to the smaller LMs trained using FL. Notably, it is usually not common to fine-tune LLMs due to the formidable computational costs and protracted training time. Therefore, we utilized in-context learning that enables direct inference from pre-trained LLMs, specifically few-shot prompting, and compared them with models trained using FL. We followed the experimental protocol outlined in a recent study^32^ and evaluated all the models on two NER datasets (2018 n2c2 and NCBI-disease) and two RE datasets (2018 n2c2, and GAD). The results, as summarized in Fig. 3, show that (1) a longer prompt with more input examples (e.g., 10-shot and 20-shot) often enhances the performance of LLMs; and (2) FL, whether implemented with a BERT-based model (BlueBERT) or GPT-based model (GPT-2), consistently outperformed LLMs by a large margin.

## Discussion

In this study, we visited FL for biomedical NLP and studied two established tasks (NER and RE) across 7 benchmark datasets. We examined 6 LMs with varying parameter sizes (ranging from BiLSTM-CRF with 20 M to transformer-based models up to 334 M parameters) and compared their performance using centralized learning, single-client learning, and federated learning. On almost all the tasks, we showed that federated learning achieved significant improvement compared to single-client learning and oftentimes performed comparably to centralized learning without data sharing, demonstrating it as an effective approach for privacy-preserved learning with distributed data. The only exception is in Table 2, where the best single-client learning model (check the standard deviation) outperformed FedAvg when using BERT and Bio_ClinicalBERT on EUADR datasets (the average performance was still left behind, though). We believe this is due to the lack of training data. As each client only owned 28 training sentences, the data distribution, although IID, was highly under-represented, making it hard for FedAvg to find the global optimal solutions. Surprisingly, FL achieved reasonably good performance even when the training data was limited (284 total training sentences from all clients), confirming that transfer learning from either the general text domain (e.g., BERT and GPT-2) or biomedical text domain (e.g., BlueBERT, BioBERT, Bio_ClinicalBERT) is beneficial to the downstream biomedical NLP task and pretraining on medical data often gives a further boost. Another interesting finding is that GPT-2 always gave inferior results compared to BERT-based models. We believe this is because GPT-2 is pre-trained on text generation tasks that only encode left-to-right attention for the next word prediction. However, this unidirectional nature prevents it from learning more about global context, which limits its ability to capture dependencies between words in a sentence.

In the sensitivity analysis of FL to client sizes, we found there is a monotonic trend that, with a fixed number of training data, FL with fewer clients tends to perform better. For example, the classical BiLSTM-CRF model (20 M), with a fixed number of total training data, performs better with few clients, but performance deteriorates when more clients join in. It is likely due to the increased learning complexity as FL models need to learn the inter-correlation of data across clients. Interestingly, the transformer-based model (≥108 M), which is over 5 sizes larger compared to BiLSMT-CRF, is more resilient to the change of federation scale, possibly owing to its increased learning capacity.

We analyzed the performance of FedProx in real-world non-IID scenarios and compared it with FedAvg to study the behavior of different FL algorithms under data heterogeneity. Although the FedProx achieved slightly better performance than FedAvg when the data were non-IID distributed, it is very sensitive to the hyper-parameter *μ,* which strikes to balance the local objective function and the proximal term. Specifically, when data was IID, and *μ* was set to a large value (e.g., *μ* = 1), FedProx yielded a 2.4% lower lenient F1-score compared to FedAvg. When the data were non-IID, this performance gap further widened to 5.4%. It is also noteworthy that when *μ* is set to 0, and all the clients are forced to perform an equal number of local updates, FedProx essentially reverts to FedAvg.

We also investigated the impact of model size on the performance of FL. We observed that as the model size increased, the performance gap between centralized models and FL models narrowed. Interestingly, BioBERT, which shares the same model architecture and is similar in size to BERT and Bio_ClinicalBERT, performs comparably to larger models (such as BlueBERT), highlighting the importance of pre-training for model performance. Overall, the size of the model is indicative of its learning capacity; large models tend to perform better than smaller ones. However, large models require longer training time and more computation resources, which results in a natural trade-off between accuracy and efficiency.

Compared with LLMs, FL models were the clear winner regarding prediction accuracy. We hypothesize that LLMs are mostly pre-trained on the general text and may not guarantee performance when applied to the biomedical text data due to the domain disparity. As LLMs with few-shot prompting only received limited inputs from the target tasks, they are likely to perform worse than models trained using FL, which are built with sufficient training data. To close the gap, specialized LLMs pre-trained on medical text data^33^ or model fine-tuning^34^ can be used to further improve the LLMs’ performance. Another interesting fact is that with more input examples (e.g., 10-shot and 20-shot), LLMs often demonstrate increased prediction performance, which is intuitive as LLMs receive more knowledge, and the performance should be increased accordingly.

While seeing many promising results of FL for LMs, we acknowledge our study suffers from the following limitations: (1) most of our experiments, excluding the non-IID study, are conducted in a simulated environment with synthetic data split, which may not perfectly align with the distribution patterns of real-world FL data. (2) we mostly focused on horizontal FL but have not extended to vertical FL^35^. (3) we have not considered FL combined with privacy techniques such as differential privacy^36^ and homomorphic encryption^37^. To address these limitations and further advance our understanding of FL for LMs, our future study will focus on the real-world implementation of FL and explore the practical opportunities and challenges in FL, such as vertical FL and FL combined privacy techniques. We believe our study will offer comprehensive insights into the potential of FL for LMs, which can serve as a catalyst for future research to develop more effective AI systems by leveraging distributed clinical data in real-world scenarios.

## Methods

### NLP tasks and corpora

We compared FL with alternative training schemes on 8 biomedical NLP datasets with a focus on two NLP tasks: NER (5 corpora) and RE (3 corpora). The NER and RE are two established tasks for information extraction in biomedical NLP. Given an input sequence of tokens, the goal of NER is to identify and classify the named entities, such as diseases and genes, present in the sequence. RE is often the follow-up task that aims to discover the relations between pairs of named entities. For example, a gene-disease relation (BRCA1-breast cancer) can be identified in a sentence: “Mutations of BRCA1 gene are associated with breast cancer”. For RE tasks, we take the entity positions as given and formulate the problem as follows: given a sentence and the spans of two entities, the task is to determine the relationship between the two entities.

We select the corpora using the following protocols: (1) Publicity. The corpora should be publicly available to ensure that the results obtained are reproducible. (2) Popularity. The corpora should be used in other well-cited papers so that the quality of the data is ensured. (3) Diversity. The corpora should represent as many as the real-world biomedical NLP tasks. A summary of the selected datasets can be found in Table 4; we defer to Supplementary Methods for more detailed descriptions of each dataset.

**Table 4:** List of corpora and their statistics

Corpus	Entity/ Relation Type	Corpora type	year	Task	Train	Dev	Test
2018 n2c2^41^	8 entities^1^	discharge summaries	2018	NER	48727	6091	6091
BC2GM^42^	gene	Medline abstract	2008	NER	26006	3251	3251
BC4CHEMD^43^	drug/chem	PubMed abstract	2015	NER	94170	11772	11771
JNLPBA^44^	gene	GENIA version 3.02 corpus	2003	NER	29559	3695	3695
NCBI-disease^45^	disease	PubMed abstract	2014	NER	10125	1266	1266
2018 n2c2^41^	disease	discharge summaries	2018	RE	72786	9099	9098
EUADR^46^	gene-disease	Medline abstracts	2012	RE	284	36	35
GAD^21^	gene-disease	genetic association studies	2004	RE	4097	513	512

The data splits are counted based on the number of sentences.

^1^A total of 8 entities are considered including reason, frequency, ADE, strength, duration, route, form, and dosage. Details about the 2018 n2c2 dataset can be found in Supplementary materials.

### Federated learning algorithms

FL represents a family of algorithms that aims to train models in a distributed environment in a collaborative manner. Consider a scenario where there are K clients with distributed data $D=\{{D}_{1},{D}_{2},...,{D}_{k}\}$, where ${D}_{i}={D}_{{X}_{i}\times {Y}_{i}}$, and ${X}_{i}$ and ${Y}_{i}$ are the input and output space, respectively. The typical FL aims to solve the optimization problem as in Eq. (1)

1 $${\sum }_{i=1}^{K}{P}_{i}{F}_{i}\left(w\right)\,{\rm{where}}\,{F}_{k}={\sum }_{j=1}^{\left|{D}_{k}\right|}{L}_{w}\left({X}_{j},{Y}_{j}\right),$$

where *w* denote the weights of the model being learned, ${F}_{i}$ is the local objective of the *i*th clients, and *p*_*i*_ is the weight of the *i*th clients such that ${p}_{i} \,>\, 0$ and ${\sum }_{i=1}^{K}{p}_{i}=1$. The weights are usually determined by the quantity of clients’ training samples. For example, it equals $\frac{1}{K}$ when clients share the same amount of training data.

In an FL game, there are two types of players: server and client. The server is the compass that navigates the whole process of FL including signaling the start and end of federated learning, synchronizing the local model updates, and dispatching the updated models. The clients are responsible for fetching models from the server, updating models using their local data, and sending the updated models back to the server.

Throughout the whole process, there are four steps: (1) the clients use their own data to optimize the local objectives—**local updates**, (2) local clients upload the updated model or gradients to the server, (3) the server acquires the local models and synchronize the updates—**model aggregation**, and (4) server dispatch the models to the clients. While different FL algorithms may have specialized designs for local updates or model aggregation, they share the same training paradigm.

We considered the two most popular FL algorithms called Federated Averaging (FedAvg)^30^ and another variant FedProx^31^. **FedAvg** is the most basic and standard FL algorithm that uses stochastic gradient descent (SGD) to progressively update the local model. More specifically, each client locally takes a fixed number of gradient descent steps on their local model using their local training data. On another hand, the server will aggregate these local models by taking the weighted average as the resulting new model for the next round. However, in FedAvg, the number of local updates can be determined by the size of the data. When the size of the data varies, the local updates performed locally can be significantly different. **FedProx** was introduced to tackle the issue of heterogeneous local updates in FedAvg. By adding a proximal term to the objective of the local update, the impact of variable local updates is suppressed. More specifically, at iteration *t*, the inner local updates are trying to find the solution that minimizes the objective, as shown in Eq. (2)

2 $${\rm{Min}}_{w}\frac{1}{{n}_{k}}{\sum }_{i=1}^{{n}_{k}}{L}_{w}\left({X}_{i},{Y}_{i}\right)+\frac{\mu }{2}\left|\left|w-{w}^{t}\right|\right|,$$

where ${w}^{t}$ is the weights of the network from iteration *t*. A comparison of FedAvg and FedProx can be found in Algorithm 1 and Algorithm 2.

#### Algorithm 1

Federated learning algorithms (FedAvg/**FedProx**)

Notation: ${X}_{i}$ indicates data from client *i*, *K* is the total number of clients, *T* is the maximum training round, *n* is the sum of ${n}_{1}$ to ${n}_{k}$, ${p}_{i}$ is the weights for the *i*th client

Initialize server model weights *w*(1)

Initialize client model weights ${w}_{i}\,\forall\, i={1,2},\ldots ,K$

For each round t = 1, 2, … T do

 Send server model weight $w(t)$ to each client

 For each client $k={1,2},\ldots ,K$ do

 Client *k* perform LocalUpdate $({X}_{k},{Y}_{k},{w}_{k})$ ← Algorithm 2

 End for

 $$w\left(t+1\right)=\mathop{\sum }\nolimits_{i=1}^{K}{p}_{i}{w}_{i}$$ ← model aggregation

End for

#### Algorithm 2

Local model training using mini-batch stochastic gradient descent (LocalUpdate) (FedAvg/**FedProx**)

Notation: R is the local update round, B is the number of batches, ${f}_{{w}_{r}}$ is the neural network parameterized by ${w}_{r}$, $\eta$ is the learning rate, $\mu$ is the hyper-parameter in FedProx

For each round $r=\mathrm{1,2},\ldots ,R$ do / Repeat until find the approximate minimizer of ${\boldsymbol{w}}\approx {\boldsymbol{argmi}}{{\boldsymbol{n}}}_{{\boldsymbol{w}}}{\boldsymbol{L}}({{\boldsymbol{f}}}_{{{\boldsymbol{w}}}_{{\boldsymbol{r}}}}({{\boldsymbol{X}}}_{{\boldsymbol{b}}}),{{\boldsymbol{Y}}}_{{\boldsymbol{b}}})$ $+\frac{{\boldsymbol{\mu }}}{{\boldsymbol{2}}}{{||}{{\boldsymbol{w}}}_{{\boldsymbol{k}}}-{{\boldsymbol{w}}}_{{\boldsymbol{k}}}\left({\boldsymbol{t}}\right){||}}^{{\boldsymbol{2}}}$

 Randomly shuffle ${X}_{k}$ and create B batches $(({X}_{1},{Y}_{1}),({X}_{2},{Y}_{2}),\ldots ,({X}_{B},{Y}_{B}))$

 $${L}_{{w}_{r}}=L({f}_{{w}_{r}}({X}_{b}),{Y}_{b})$$ $${\boldsymbol{+}}\frac{{\boldsymbol{\mu }}}{{\boldsymbol{2}}}{{||}{{\boldsymbol{w}}}_{{\boldsymbol{k}}}{\boldsymbol{-}}{{\boldsymbol{w}}}_{{\boldsymbol{k}}}\left({\boldsymbol{t}}\right){||}}^{{\boldsymbol{2}}}\,$$

 For each mini-batch $b=\mathrm{1,2},\ldots ,B$ do

 $${w}_{r+1}={w}_{r}-\eta {\nabla L}_{{w}_{r}}({X}_{b},{Y}_{b})$$

End for

### Study design

**Fig. 4:**
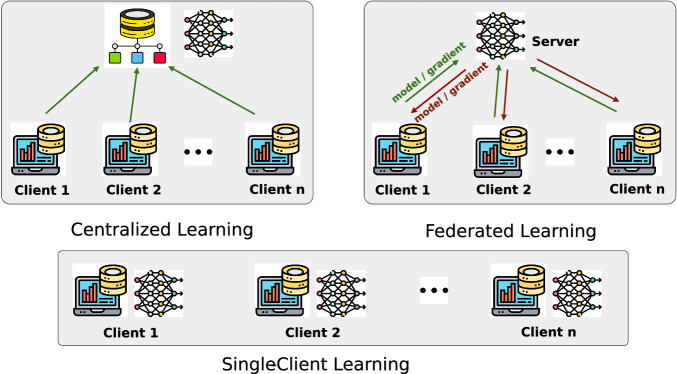
A comparison of centralized learning, federated learning, and single-client learning. The arrows indicate the data flow through the model training process.

As shown in Fig. 4, we explored three learning methods: (1) federated learning, centralized learning, and single-client learning. To simulate the conventional learning scenario, we varied the data scale and conducted the following experiments: centralizing all client data to train a single model (centralized learning) and training separate models on each client’s local data (single-client learning).

#### Models

To better understand the effect of LMs on FL, we chose models with various sizes of parameters from 20 M to 334 M, including Bidirectional Encoder Representations from Transformer (BERT)^1^, and Generative Pre-trained Transformer (GPT)^38^, as well as classical RNN-based model like BiLSTM-CRF^39^. BERT-based models utilize a transformer encoder and incorporate bi-directional information acquired through two unsupervised tasks as a pre-training step into its encoder. Different BERT models differ in their pre-training source dataset and model size, deriving many variants such as BlueBERT^12^, BioBERT^8^, and Bio_ClinicBERT^40^. BiLSTM-CRF is the only model in our study that is not built upon transformers. It is a bi-directional model designed to handle long-term dependencies, is used to be popular for NER, and uses LSTM as its backbone. We selected this model in the interest of investigating the effect of federation learning on models with smaller sets of parameters. For LLMs, we selected GPT-4, PaLM 2 (Bison and Unicorn), and Gemini (Pro) for assessment as both can be publicly accessible for inference. A summary of the model can be found in Table 5, and details on the model description can be found in Supplementary Methods.

**Table 5:** List of LMs used for comparison

Model	Param	Backbone	Pre-trained source	Year
BiLSTM-CRF^39^	20 M	LSTM	–	2015
BERT^1^	109 M	Transformer encoder	Wikipedia + BooksCorpus	2018
BlueBERT^12^	334 M	Transformer encoder	PubMed	2019
BioBERT^8^	108 M	Transformer encoder	Wikipedia + BooksCorpus + PubMed + PMC	2020
Bio_ClinicalBERT^9^	108 M	Transformer encoder	Clinical notes	2019
GPT-2^38^	124 M	Transformer decoder	Wikipedia +news+books	2019
GPT-4^47^	–	Transformer decoder	–	2023
PaLM 2^48^	–	Transformer	Web documents, books, code, mathematics, and conversational data	2023
Gemini^49^	–	Transformer decoder	Web documents, books, code, images, audio, and video data	2023

### Training details

#### Data preprocessing

we adapted most of the datasets from the BioBERT paper with reasonable modifications by removing the duplicate entries and splitting the data into the non-overlapped train (80%), dev (10%), and test (10%) datasets. The maximum token limit was set at 512, with truncation—coded sentences with lengths larger than 512 were trimmed.

#### Federated learning simulation

We considered two different learning settings: learning from IID data and learning from non-IID data. For the first setting, we randomly split the data into k folds uniformly. For most of our experiments, k was chosen as 10, while we also varied k from 2 to 10 to study the impact of the size of the federation. For the second setting, we considered learning from heterogeneous data collected from different sources. This represents the real-world scenario where complex and entangled heterogeneities are co-existed. We picked BC2GM and JNLPBA as two independent clients, both targeting the same gene entity recognition tasks but were collected from different sources. To show that they are non-IID distributed, we have conducted data distribution analysis (i.e., calculate the distribution distance and plot t-SNE on embedded features space), which can be found in Supplementary Discussions.

#### LLMs with few-shot prompting

**Fig. 5:**
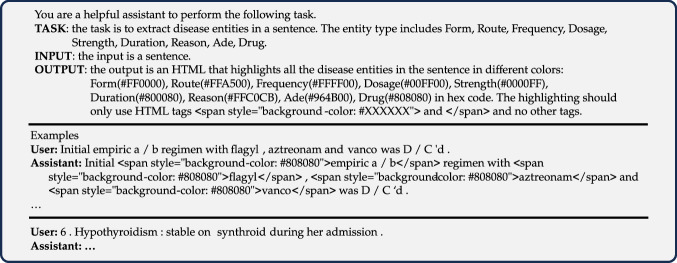
An example of applying few-shot prompting in an LLM to solve an NER task. We formulated the prompt to include a description of the task, a few examples of inputs (i.e., raw texts) and outputs (i.e., annotated texts), and a query text at the end.

We followed a similar experiment protocol as in the previous study^32^. Figure 5 shows an example of applying few-shot prompting in a LLM to solve an NER task. A RE task can be solved similarly by changing the task description, and input-output pairs. Notably, we simulate 1-/5-/10-/20-shot prompting by varying the number of input examples that are randomly selected from the training dataset. For model evaluation, we randomly selected 200 test samples in the test dataset and reported the prediction performance over the selected samples.

#### Training models

For models that require training, we used Adam to optimize our models with an initial learning rate of 0.001 and momentum of 0.9. The learning rate was scheduled by *linear_scheduler_with_warmup*. All experiments were performed on a system equipped with an NVIDIA A100 GPU and an AMD EPYC 7763 64-core Processor.

#### Reported evaluation

For NER, we reported the performance of these metrics at the macro average level with both strict and lenient match criteria. Strict match considers the true positive when the boundary of entities exactly matches with the gold standard, while lenient considers true positives when the boundary of entities overlaps between model outputs and the gold standard. For all tasks, we repeated the experiments three times and reported the mean and standard deviation to account for randomness.

### Reporting summary

Further information on research design is available in the Nature Research Reporting Summary linked to this article.

### Supplementary information

Reporting Summary

supplemental materials

## Data Availability

All the datasets involved in this study are publicly available from the following official websites: 2018 n2c2: https://portal.dbmi.hms.harvard.edu/projects/n2c2-nlp/. BC2GM: https://biocreative.bioinformatics.udel.edu/tasks/. BC4CHEMD: https://biocreative.bioinformatics.udel.edu/resources/biocreative-iv/chemdner-corpus/. JNLPBA: http://www.geniaproject.org/shared-tasks/bionlp-jnlpba-shared-task-2004. NCBI-disease: https://www.ncbi.nlm.nih.gov/CBBresearch/Dogan/DISEASE/. EUADR: https://biosemantics.erasmusmc.nl/index.php/resources/euadr-corpus. GAD: https://maayanlab.cloud/Harmonizome/dataset/GAD+Gene-Disease+Associations.

## Code availability

Our project codes are publicly available on Github: Train and evaluate FL models: https://github.com/PL97/FedNLP. Texts preprocessing: https://github.com/PL97/Brat2BIO. Evaluation: https://github.com/PL97/NER_eval. LLMs evaluations: https://github.com/GaoxiangLuo/LLM-BioMed-NER-RE.
