# Supplementary material for: An in-depth evaluation of federated learning on biomedical natural language processing for information extraction
Source: NPJ Digit Med. 2024 May 15;7:127. doi: 10.1038/s41746-024-01126-4 (PMC11096157; doi:10.1038/s41746-024-01126-4)
Supplement: Supplementary file 1 — Reporting Summary [file 41746_2024_1126_MOESM1_ESM.pdf]

nature portfolio

Corresponding author(s): Rui Zhang, Ju Sun

Last updated by author(s): Apr 4, 2024

Reporting Summary

Nature Portfolio wishes to improve the reproducibility of the work that we publish. This form provides structure for consistency and transparency in reporting. For further information on Nature Portfolio policies, see our Editorial Policies and the Editorial Policy Checklist.

Statistics

For all statistical analyses, confirm that the following items are present in the figure legend, table legend, main text, or Methods section.

n/a	Confirmed	
☒	☐	The exact sample size (*n*) for each experimental group/condition, given as a discrete number and unit of measurement
☒	☐	A statement on whether measurements were taken from distinct samples or whether the same sample was measured repeatedly
☒	☐	The statistical test(s) used AND whether they are one- or two-sided<br>*Only common tests should be described solely by name; describe more complex techniques in the Methods section.*
☒	☐	A description of all covariates tested
☒	☐	A description of any assumptions or corrections, such as tests of normality and adjustment for multiple comparisons
☐	☒	A full description of the statistical parameters including central tendency (e.g. means) or other basic estimates (e.g. regression coefficient) AND variation (e.g. standard deviation) or associated estimates of uncertainty (e.g. confidence intervals)
☒	☐	For null hypothesis testing, the test statistic (e.g. *F*, *t*, *r*) with confidence intervals, effect sizes, degrees of freedom and *P* value noted<br>*Give P values as exact values whenever suitable.*
☒	☐	For Bayesian analysis, information on the choice of priors and Markov chain Monte Carlo settings
☒	☐	For hierarchical and complex designs, identification of the appropriate level for tests and full reporting of outcomes
☒	☐	Estimates of effect sizes (e.g. Cohen's *d*, Pearson's *r*), indicating how they were calculated

Our web collection on statistics for biologists contains articles on many of the points above.

Software and code

Policy information about availability of computer code

Data collection	No commercial software were used. All open-source and custom codes were provided in Github
Data analysis	No commercial software were used. All open-source and custom codes were provided in Github

For manuscripts utilizing custom algorithms or software that are central to the research but not yet described in published literature, software must be made available to editors and reviewers. We strongly encourage code deposition in a community repository (e.g. GitHub). See the Nature Portfolio guidelines for submitting code & software for further information.

Data

Policy information about availability of data

All manuscripts must include a data availability statement. This statement should provide the following information, where applicable:

- Accession codes, unique identifiers, or web links for publicly available datasets
- A description of any restrictions on data availability
- For clinical datasets or third party data, please ensure that the statement adheres to our policy

All the datasets involved in this study are publicly available from the following official websites:
2018 n2c2:https://portal.dbmi.hms.harvard.edu/projects/n2c2-nlp/
BC2GM:https://biocreative.bioinformatics.udel.edu/tasks/
BC4CHEMD:https://biocreative.bioinformatics.udel.edu/resources/biocreative-iv/chemdner-corpus/

JNLPBA:http://www.geniaproject.org/shared-tasks/bionlp-jnlpba-shared-task-2004
NCBI-disease:https://www.ncbi.nlm.nih.gov/CBBresearch/Dogan/DISEASE/
EUADR: https://biosemantics.erasmusmc.nl/index.php/resources/euadr-corpus
GAD:https://maayanlab.cloud/Harmonizome/dataset/GAD+Gene-Disease+Associations

Research involving human participants, their data, or biological material

Policy information about studies with human participants or human data. See also policy information about sex, gender (identity/presentation), and sexual orientation and race, ethnicity and racism.

Reporting on sex and gender	this information has not been collected
Reporting on race, ethnicity, or other socially relevant groupings	this information has not been collected
Population characteristics	this information has not been collected
Recruitment	this study has no recruitment of subjects
Ethics oversight	This study used de-identified public data, thus no ethics oversights was applied

Note that full information on the approval of the study protocol must also be provided in the manuscript.

Field-specific reporting

Please select the one below that is the best fit for your research. If you are not sure, read the appropriate sections before making your selection.

☐ Life sciences ☒ Behavioural & social sciences ☐ Ecological, evolutionary & environmental sciences

For a reference copy of the document with all sections, see nature.com/documents/nr-reporting-summary-flat.pdf

Behavioural & social sciences study design

All studies must disclose on these points even when the disclosure is negative.

Study description	We conducted an evaluation of federated learning in the biomedical NLP with a application of information extraction from biomedical corpora
Research sample	We used 7 public datasets containing biomedical literatures and other text data. Details of the dataset can be found in the method section and data availability section
Sampling strategy	Data were randomly sampled into train (80%), dev (20%), and test (10%) sets.
Data collection	NA - no collection was conducted in this study
Timing	Please refer to Method section to see the time of collection for each corpus
Data exclusions	We removed duplicated entries
Non-participation	NA - no recruitment involved
Randomization	The data were randomly splited into train, dev, test sets. Experiments were repeated for three times to account for randomness

Reporting for specific materials, systems and methods

We require information from authors about some types of materials, experimental systems and methods used in many studies. Here, indicate whether each material, system or method listed is relevant to your study. If you are not sure if a list item applies to your research, read the appropriate section before selecting a response.

Materials & experimental systems

n/a	Involved in the study
☒	☐ Antibodies
☒	☐ Eukaryotic cell lines
☒	☐ Palaeontology and archaeology
☒	☐ Animals and other organisms
☒	☐ Clinical data
☒	☐ Dual use research of concern
☒	☐ Plants

Methods

n/a	Involved in the study
☒	☐ ChIP-seq
☒	☐ Flow cytometry
☒	☐ MRI-based neuroimaging

Plants

Seed stocks	this study does not involves any plant object, thus the information is not applicable
Novel plant genotypes	this study does not involves any plant object, thus the information is not applicable
Authentication	this study does not involves any plant object, thus the information is not applicable
