# Supplementary material for: An in-depth evaluation of federated learning on biomedical natural language processing for information extraction
Source: NPJ Digit Med. 2024 May 15;7:127. doi: 10.1038/s41746-024-01126-4 (PMC11096157; doi:10.1038/s41746-024-01126-4)
Supplement: Supplementary file 2 — supplemental materials [file 41746_2024_1126_MOESM2_ESM.pdf]

Supplementary Figures

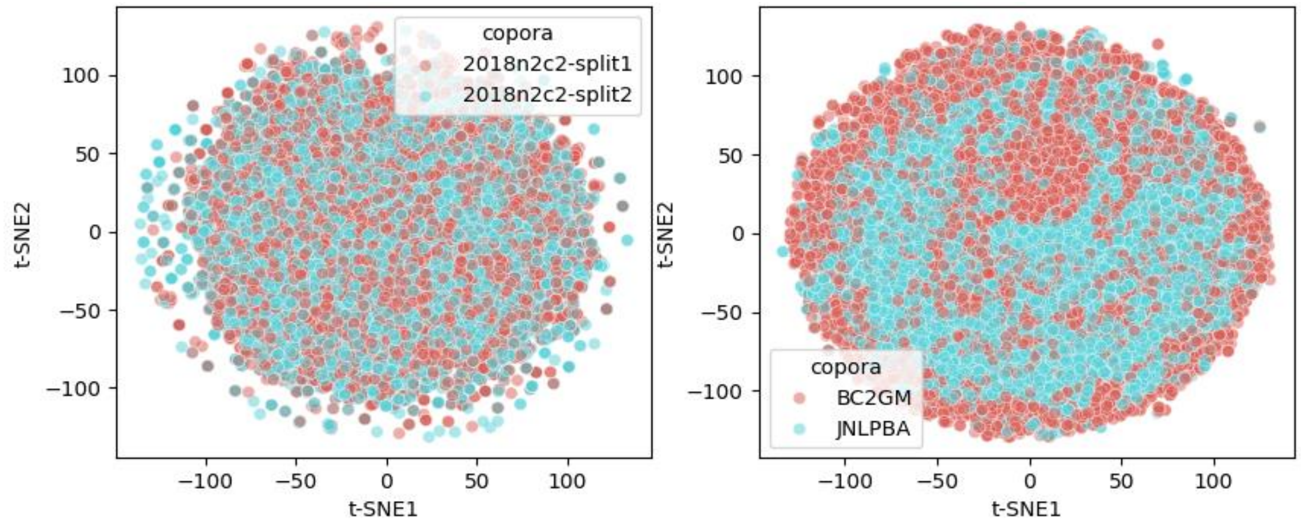

Supplementary Fig. 1 t-SNE visualization on IID (left) and non-IID (right) data

Supplementary Tables

Supplementary Table 1. Comparison of LLM and FL on NER tasks. We report the F1-score measured by lenient and strict (in parenthesis) matching scheme[1].

Corpus	Model	1-shot	5-shot	10-shot	20-shot	s/instance
2018 n2c2	GPT-4	0.677 (0.475)	0.750 (0.582)	0.752 (0.589)	0.749 (0.616)	8.24
	PaLM Bison	0.389 (0.248)	0.580 (0.441)	0.652 (0.519)	0.653 (0.544)	2.21
	PaLM Unicorn	0.638 (0.474)	0.740 (0.584)	0.749 (0.621)	0.700 (0.565)	5.45
	Gemini Pro	0.344 (0.233)	0.571 (0.442)	0.648 (0.504)	0.694 (0.566)	2.25
	BlueBERT (FL)	**0.899 (0.824)**				1.49×10^{-3}
	GPT-2 (FL)	0.840 (0.784)				$\mathbf{6.12 \times 10^{-4}}$

NCBI	GPT-4	0.814 (0.625)	0.816 (0.658)	0.820 (0.703)	0.834 (0.722)	4.33
	PaLM Bison	0.692 (0.577)	0.678 (0.596)	0.702 (0.601)	0.756 (0.640)	1.30
	PaLM Unicorn	0.848 (0.729)	0.822 (0.723)	0.782 (0.696)	0.807 (0.726)	2.96
	Gemini Pro	0.655 (0.524)	0.616 (0.507)	0.650 (0.568)	0.779 (0.654)	1.24
	BlueBERT (FL)	**0.986 (0.954)**				1.49x10^{-3}
	GPT-2 (FL)	0.868 (0.830)				**6.12x10^{-4}**

[1] The highest scores for each corpus are bold, and the best performing LLM results are underlined.

Supplementary Table 2. Comparison of LLM and FL on RE tasks using F1-score[1].

Corpus	Model	1-shot	5-shot	10-shot	20-shot	instance/
2018 n2c2	GPT-4	0.607	0.745	0.852	0.882	0.935
	PaLM Bison	0.301	0.319	0.408	0.319	0.478
	PaLM Unicorn	0.851	0.888	0.848	0.866	1.14
	Gemini Pro	0.213	0.276	0.306	0.412	0.463
	BlueBERT (FL)	**0.969**				2.61x10^{-2}
	GPT-2 (FL)	0.946				**1.58x10^{-2}**
GAD	GPT-4	0.421	0.400	0.542	0.543	0.738
	PaLM Bison	0.454	0.448	0.456	0.469	0.453
	PaLM Unicorn	0.481	0.487	0.539	0.550	0.810
	Gemini Pro	0.460	0.462	0.542	0.513	0.393
	BlueBERT (FL)	**0.742**				2.61x10^{-2}
	GPT-2 (FL)	0.721				**1.58x10^{-2}**

[1] The highest scores for each corpus are bold, and the best performing LLM results are underlined.

Supplementary Table 3. The measure of data distribution shift using 2-Wasserstein distance

corpora	IID setting		non-IID setting	
	2018 n2c2 (split 1)	2018 n2c2 (split 2)	BC2GM	JNLPBA
Mean norm	16.02	16.07	15.15	15.06
Variance norm	17.57	16.88	24.35	17.23
2-Wasserstein distance	**3.95x10^-2**		**3.40**	

Supplementary Methods

Below is a detailed description of the datasets used in the main paper.

- **2018 National NLP Clinical Challenges (n2c2) Shared Task**: 2018 n2c2 corpus contains 505 discharge summaries from the MIMIC-III clinical care database. The goal of the task is to extract entity tags (*reason, frequency, ADE, strength, duration, route, form, drug, and dosage*) that indicate the presence of drug and ADE information, and relations (*strength-drug, duration-drug, route-drug, form-drug, ADE-drug, Dosage-drug, reason-drug, and frequency-drug*) between the entities.
- **BioCreative II Gene Mention Recognition (BC2GM)**: BC2GM Dataset collected text data related to gene information. The dataset comprises a set of sentences, and a set of gene mentions (GENE annotations) for each sentence. Some GENE annotations in a sentence may also have alternate boundaries that are judged by

human annotators which can be essentially equivalent references (ALTGENE annotations). The goal of the task is to identify gene mentions in a sentence according to its start and end characters**.**

- **BioCreative IV Chemical Compound and Drug Name Recognition (BC4CHEMD)**: BC4CHEMD contains a total of 84,355 chemical mention annotations from 10,000 PubMed abstracts which are manually labeled by some chemistry literature experts. The goal of the task is to classify the text into multiple CEM classes: *systematic, identifiers, formula, trivial, abbreviation, family, and multiple.*
- **JNLPBA**: JNLPBA originated from the GENIA version 3.02. It is a selection of 2,000 abstracts with a controlled search on MEDLINE using the Medical Subject Headings (MeSH) terms *'human'*, *'blood cells'*, and *'transcription factors'*. The abstracts were hand-annotated to 36 terminal classes according to a small taxonomy of 48 classes based on a chemical classification.
- **NCBI-disease**: The NCBI-disease corpus is collected from 793 PubMed abstracts that are fully annotated at the disease mentions and concept level based on corresponding identifiers from either MeSH or Online Mendelian Inheritance in Man (OMIM). It includes 6892 disease mentions, which are mapped to 790 unique disease concepts. 12% link to an OMIM identifier, while the remaining contain a MeSH identifier. In addition, 91% of mentions are described as a single disease concept, while the remaining link to a combination of concepts.

- **EUADR**: EUADR corpus was annotated for disorders, drugs, genes, and their inter-relationships. Three experts were used to annotate a set of 100 abstracts for each of the drug-disorder, drug-target, and target-disorder relations. The drug-disorder and drug-target relations were composed of 100 randomly selected abstracts from the PubMed result. For the target-disorder set, 50 abstracts were randomly selected from gene disorder, and 50 abstracts were randomly selected from SNP-disorder relation.
- **Gene Associations Database (GAD)**: Gene Associations Dataset is a corpus that provides a public, comprehensive repository of molecular, clinical, and study parameters for > 5000 human genetic association studies to explore gene-disease relations. It contains 10697 genes, 12774 diseases, and 74928 gene-disease associations.

Below are detailed descriptions of LMs and LLMs used in the main paper.

- **BERT**: Bidirectional Encoder Representations from Transformer (BERT) was developed in 2018 by researchers at Google. The BERT's model architecture is a multi-layer bidirectional Transformer encoder. It employs encoders as a sub-structure to pre-training models for NLP tasks. BERT comprehends language via Masked Language Modeling (MLM) and Next Sentence Prediction (NSP) mechanisms. By assuming a blinder with MLM, BERT learns bidirectional contexts within sentences. The model takes random sentences as input, masks

certain words, and then reconstructs the masked words from the surrounding text. BERT's ability to process two sentences simultaneously and determine if the second follows the first enables it to achieve NSP, facilitating the maintenance of long-distance relationships between texts. The BERT was pre-trained on English Wikipedia (2.5B words) and BookCorpus (800M words). BERT has two models. The BERT_BASE has 12 layers, 768 widths, 12 heads, and a total of 110M parameters. The BERT_LARGE has 24 layers, 1024 widths, 16 heads, and a total of 340M parameters.

- **GPT-2**: Generative Pretrained Transformer 2 (GPT2) was developed by OpenAI researchers in 2019. GPT-2 models language using transformer decoders. GPT-2 is designed for predicting the next sentence in sentences. It achieves this using the architecture of GPT-1 with an extra normalization layer to the input of each sub-block and after the final self-attention layer. GPT-2's input includes texts' weight embeddings and their positional embeddings for context extraction. The input is then passed through the multi-head attention layer in the transformer decoder blocks, followed by a feed-forward layer. Finally, the softmax outputs a probability distribution. The GPT-2 was trained on an extensive corpus of WebText (40 GB of text, 8 million documents, from 45 million webpages upvoted on Reddit). It has 48 layers, 1600 widths, and 1.5B parameters.
- **BI-LSTM-CRF**: The Bidirectional LSTM CRF network combines a bidirectional LSTM network with a CRF network. It uses both long-distance past and future

input features via the bidirectional LSTM layer and sentence-level tag information via the CRF layer. The initial layer of the model, designed to capture the semantics of the input text sequence, is a Bi-LSTM network. This layer's output is then fed into a CRF layer, which generates a probability distribution over the tag sequence by utilizing the interdependencies among the labels of the entire sequence.

- **GPT-4**: The Generative Pretrained Transformer 4 (GPT-4) was released by OpenAI in 2023 without disclosure of model architecture, training, dataset, etc. Different from GPT-3[1], GPT-4 was fine-tuned using Reinforcement Learning from Human Feedback (RLHF). The checkpoint used for experiments in this paper is GPT-4-0613.
- **PaLM 2**: The Pathways Language Model (PaLM) 2 is built upon the Transformer. Specific information regarding its model size and architecture has not been shared in public. From available information, however, the predecessor of PaLM 2, known as PaLM[2], has 540B parameters, and deploys a standard Transformer architecture in a decoder-only configuration, albeit with certain alterations (e.g., SwiGLU Activation, RoPE embedding, etc). The checkpoints used for experiments in this paper are "unicorn-001" and "bison-002", the largest two models in the PaLM 2 family.
- **Gemini**: The family of multimodal models, Gemini, was released by Google in 2023. These models are built upon Transformer decoders to output text and

images, and allow interleaved sequences of text, image, video, and audio as inputs. Details regarding the size of the two largest models in this family remain undisclosed. The training of these models encompasses a diverse dataset comprising web documents, books, and code, along with image, audio, and video data, though specific details about this data are not publicly available. The checkpoint used for experiments in this paper is Gemini-1.0-pro.

Supplementary Discussion

This section provides supplemental discussion results on LLMs comparision and data distribution shift analysis.

LLMs comparison. Supplementary Table 1 and Supplementary Table 2 present the NER and RE performance measures of selected pretrained LLMs with few-shot prompting, and compared with BlueBERT and GPT-2 trained using FL, as measured by F1-score with strict and lenient matching scheme. Moreover, we calculate the running time of different models measured by seconds of processing one instance (s/instance). Notably, all the LLMs are called via API thus the communication cost of transferring data between the local server and remote cloud that hosts LLMs are also included. From the results, it is clear that small models trained using FL, either on NER or RE tasks, significantly outperform pre-trained LLMs with few-shot prompting both in terms of prediction accuracy and inference speed. Among all the selected LLMs, GPT-4 and

PaLM Unicorn often give the best-performing results while the runtime is relatively slow compared with other LLMs counterparts.

Data heterogeneity analysis. To get a better understanding of how data are distributed in our IID and non-IID simulated experiments. We conduct the following experiments to qualitatively measure the distribution distance between the two selected corpora.

Considering that text is high-dimensional data, it is hard to directly assess the similarities between two distributions. We use pre-trained language models (e.g., BERT) to extract features from texts which helps to reduce the dimension of the features and make it easy for us to analyze the distribution characteristics. We consider the two following approaches to access the distribution distance: **1) calculate the Wasserstein distance (norm of 2) to quantify the distribution distance.** We assume the encoded features from BERT follow a Gaussian distribution, denoted as $N_1(\mu_1, \Sigma_1)$ and $N_1(\mu_1, \Sigma_1)$, thus the distance between the two distributions can be quantified by Wasserstein distance with the norm of 2, as shown in Supplementary equation (1)

$$d(N_1, N_2)^2 = ||\mu_1 - \mu_2||^2{}_2 + tr\left(\Sigma_1 + \Sigma_2 - 2(\Sigma_1 \Sigma_2)^{\frac{1}{2}}\right), \tag{1}$$

Where μ_1 and μ_2 are the mean of the two Gaussian distributions Σ_1 and Σ_2 are the covariance matrix. By doing so, we can obtain the distribution distance of the two corpora, and the results are summarized in Supplementary Table 3. We can tell from the table that in the IID setting, the two distributions exhibit close mean and variance, and

the resulting 2-Wasserstein distance is also close. In contrast, in the non-IID setting, although the mean is close, the variance differs, making the distance much larger than in the IID setting. **2) use t-SNE to visualize the encoded features to see the separation.** We *sampled the same number of data points for two settings* and plotted the t-SNE visualization shown in Supplementary Fig. 1. Notably, the two corpora, represented by distinct colors, display more clear separation when the data is non-IID, forming tightly clustered groups in the visualization (right plot). Conversely, in the case of IID-distributed data, the points scatter randomly, resulting in increased overlap between the two corpora (left plot).

Supplementary References

1. Brown, T. *et al.* Language Models are Few-Shot Learners. in *Advances in Neural Information Processing Systems* vol. 33 1877–1901 (Curran Associates, Inc., 2020).
2. Chowdhery, A. *et al.* PaLM: Scaling Language Modeling with Pathways. Preprint at https://doi.org/10.48550/arXiv.2204.02311 (2022).
